# Clinical and Immunological Profile of Pediatric-Onset Systemic Lupus Erythematosus in Eastern India: A Prospective Observational Study

**DOI:** 10.7759/cureus.66709

**Published:** 2024-08-12

**Authors:** Soumya Mishra, Jyoti Ranjan Behera, Amit R Rup, Sanjay Kumar Sahu, Arun K Dash, Rama Krushna Gudu, Seba Ranjan Biswal

**Affiliations:** 1 Pediatrics, Institute of Medical Sciences and SUM Hospital, Bhubaneswar, IND; 2 Pediatrics, Kalinga Institute of Medical Sciences, Bhubaneswar, IND

**Keywords:** outcome, autoantibodies, sledai score, lupus nephritis, pediatric sle

## Abstract

Background: Systemic lupus erythematosus (SLE) is a multisystem autoimmune disease with a strong female predisposition. pSLE often results in a worse prognosis compared to adult SLE. Studies on pSLE from the Indian subcontinent are scarce.

Objective: This study aims to describe the clinical manifestations, laboratory and serological parameters, management, and outcomes of pSLE patients from a premier tertiary care institute in Eastern India.

Methods: This prospective observational study was conducted at Kalinga Institute of Medical Sciences, Bhubaneswar, from September 2020 to October 2023. Children aged 1-14 years fulfilling the Systemic Lupus International Collaborating Clinics criteria for SLE were included. A detailed history, clinical examination, and laboratory investigations were performed. Data on complications, treatment, and outcomes were collected. Statistical analysis was done using SPSS Statistics version 21 (IBM Corp. Released 2012. IBM SPSS Statistics for Windows, Version 21.0. Armonk, NY: IBM Corp.).

Results: Out of 114,009 patients (outdoor and indoor), 40 were diagnosed with pSLE, resulting in an incidence of 0.35 per 1000 children. The female-to-male ratio was 7:1. The mean age of presentation was 11.67 ± 2.37 years. Among the predominant symptoms observed, mucocutaneous manifestations were seen in 39 (97.5%), followed by pallor in 36 (90%), and fever in 33 (82.5%). The most common organ system involved was mucocutaneous, observed in 39 (97.5%) patients, followed by hematological in 36 (90%) and renal in 19 (47.5%). Lupus nephritis was observed in 19 (47.5%) patients, with class IV being the most common. Anti-nuclear antibody and anti-double-stranded DNA were positive in 39 (97.5%) and 27 (68%) of children, respectively. Complete remission was achieved in 14 (35%), improvement in 16 (40%), and flare-ups in 3 (7.5%) patients.

Conclusion: pSLE is an uncommon but severe autoimmune disease with significant multi-system involvement. Early identification and prompt treatment are crucial to minimizing adverse outcomes. This study provides detailed insights into the clinical and immunological profile of pSLE in Eastern India, underscoring the need for larger multicentric studies with long-term follow-ups.

## Introduction

Systemic lupus erythematosus (SLE) is a multisystem autoimmune disease of unknown etiology with varying severity and a strong female predisposition. Pediatric-onset SLE (pSLE) has a prevalence of one to six per 1 lakh population, whereas up to 20% of all SLE patients are diagnosed prior to 16 years of age [1]. pSLE is known to confer a worse prognosis compared to adults in terms of disease activity, organ damage, and mortality [2]. A wide spectrum of presentations of SLE depends upon genetic, geographic, and environmental factors [3]. Various studies previously have described the clinicopathological profile, management, and outcome of pediatric SLE from different regions of the world, but such studies from the Indian subcontinent are scarce [4,5]. In recent years, pSLE cases have been detected early due to increased awareness and the widespread availability of investigations. Treatment protocols have been updated recently, and most centers are providing prompt intensive treatment with regular follow-up [6]. This study is intended to describe the clinical manifestations, serological and laboratory parameters, management, and outcome of patients with pSLE from a premier tertiary care teaching hospital in Eastern India.

## Materials and methods

This prospective observational study was conducted in the Department of Pediatrics of Kalinga Institute of Medical Sciences, Bhubaneswar, between September 2020 and October 2023, after approval of the Institutional Ethics Committee of Kalinga Institute of Medical Sciences (approval number: KIIT/KIMS/IEC/215). For all SLE-suspected children between 1 and 14 years of age (outdoor and indoor), the Systemic Lupus International Collaborating Clinics (SLICC) criteria was applied [7]. Children fulfilling SLICC criteria were included in the study after obtaining informed consent from the caregiver. However, children with features of other connective tissue diseases, overlap syndrome, or already diagnosed cases of SLE on treatment were excluded.

A detailed history and complete clinical examination were performed on enrolled children, as per the predesigned structured proforma. Demographic features, including family history, clinical and laboratory parameters, manifestations at the time of diagnosis, and complications, were recorded. All patients were subjected to baseline hematological, immunological, and relevant radiological investigations as indicated. Data related to disease complications, including renal biopsy findings, were collected during the course of the disease. The time of diagnosis was defined as the time when a patient satisfied the SLICC criteria for SLE. Clinical evidence of nephritis was documented by the presence of proteinuria (≥0.5 gram/24 hours) or active urine sediment (>8 to 10 erythrocytes per high power field or casts). The histopathology of the renal biopsy in patients with lupus nephritis was classified according to the modified WHO classification [8]. Auto-immune hemolytic anemia was diagnosed by a positive Coomb’s test. Leukocytopenia was considered when the total leucocyte count was less than 4000/cmm3, and thrombocytopenia was defined as a platelet count <100,000/m3 in the absence of any offending drugs. Pleural effusion and pericardial effusion were diagnosed clinically, as were radiological and echocardiographic findings. The immunological parameters included anti-nuclear antibodies (ANA), the ANA profile, anti-double-stranded DNA (anti-ds-DNA), C3, and C4. ANA was determined by the immunofluorescence technique using Hep-2 cells as a substrate. The ANA profile was measured by the ELISA method. Complements were measured by nephelometry. The relationship between different autoantibodies and systemic involvement was assessed. Disease activity was assessed using the SLE Disease Activity Index (SLEDAI) score at admission, after two weeks, and at three months of follow-up [9]. An increase in SLEDAI score ≥3 was stamped as a flare, whereas a reduction of a score of 3 was an improvement, and a score of 0 was a remission.

Data were recorded in a Microsoft Excel sheet (Microsoft Corporation, Redmond, CA, USA) and were analyzed using SPSS Statistics version 21 (IBM Corp. Released 2012. IBM SPSS Statistics for Windows, Version 21.0. Armonk, NY: IBM Corp.). Continuous variables were presented with either a mean or standard deviation or with a median and an interquartile range. Categorical variables were presented with frequency (in percentage) and a 95% confidence interval. Mean with standard deviation and median with range were used to describe scale data according to the shape of the distribution. The chi-square test was used for categorical variables, and a p-value of <0.05 was considered statistically significant.

## Results

Out of 114,009 total patients visiting the hospital (outdoor and indoor) during the study period, 40 were diagnosed with SLE, which makes an incidence of 0.35 cases per 1000 children coming to the hospital. The majority were female, with a female-to-male ratio of 7:1. Thirty-three patients (82.5%) were between 11 and 14 years of age at diagnosis, with a mean age of onset of symptoms of 11.67 ± 2.37 years.

Among the predominant symptoms observed, mucocutaneous manifestations were seen in 39 (97.5%), followed by pallor in 36 (90%) and fever in 33 (82.5%). Clinical manifestations and their frequency of occurrence have been described in Table 1.

**Table 1:** Clinical manifestations in patients with SLE (n=40) SLE: systemic lupus erythematosus

Clinical features	No. of patients	Percentage (%)
Fever	33	82.5
Pallor	36	90.0
Lymphadenopathy	11	27.5
Weight loss	3	7.5
Serositis	15	37.5
Arthritis	19	47.5
Hepatitis	3	7.5
Hepatosplenomegaly	6	15
Hypertension	6	15
Mucocutaneous	39	97.5
Vision loss	3	7.5
Neuropsychiatric	9	22.5

The most common organ system involved was mucocutaneous, observed in 39 (97.5%) patients, followed by hematological in 36 (90%) and renal in 19 (47.5%). Various organ systems and their involvement (in percentage) are depicted in Table 2.

**Table 2:** Systemic distributions of SLE (n=40) CNS: central nervous system, SLE: systemic lupus erythematosus

System involved	No of patients	Percentage (%)
CNS	9	22.5
Cardiovascular system	10	25
Respiratory	13	32.5
Gastrointestinal	19	47.5
Renal	19	47.5
Hematological	36	90.0
Mucocutaneous	39	97.5
Locomotory	19	47.5
Eyes	04	10
Endocrine	01	2.5
Constitutional	40	100

Among mucocutaneous features, malar rash and oral ulcer were seen in 28 (70%) and were the more prevalent signs, followed by alopecia in 17 (42.5%) and photosensitivity in 16 (40%). Thirty-six children (90%) were found anemic, with combs positivity found in 10 (25%). Leukopenia and thrombocytopenia were observed in nine (22.5%) and eight (20%) children, respectively.

Out of 19 patients with lupus nephritis, 17 underwent a renal biopsy. On histology, class IV nephritis was the most common finding in seven (41.2%). Central nervous system (CNS) manifestations (headache, seizure, psychosis, stroke, and neuropathy) were found in nine (22.5%) children. Ten children (25%) had cardiovascular system involvement with features like hypertension, congestive cardiac failure, and pericardial effusion. Complement level was low in 33 (82.5%) children.

ANA and anti-ds-DNA were positive in 39 (97.5%) and 27 (68%) children, respectively. Table 3 describes various autoantibodies found in the patients and their relationship with organ involvement.

**Table 3:** Relationship between immunological profile and systemic manifestations

Antibodies	Lupus nephritis (N=19) (p-value)	Neuropsychiatric symptoms (N=9) (p-value)	Hematological (N=36) (p-value)	Mucocutaneous (N=39) (p-value)	Musculoskeletal (N=19) (p-value)	Serositis (N=15) (p-value)
Anti-ds-DNA n=27 (68%)	17 (0.005)	4 (0.09)	23 (0.15)	26 (0.48)	12 (0.58)	7 (0.03)
Anti-Smith n=12 (30%)	6 (0.84)	5 ((0.06)	9 (0.04)	11 (0.12)	5 (0.63)	4 (0.72)
Anti-nucleosome n=4 (10%)	2 (0.91)	0 (0.26)	1 (0.001)	3 (0.002)	1 (0.34)	0 (<0.001)
Anti-ribonuceoprotein n=5 (12%)	2 (0.72)	4 (0.001)	2 (<0.001)	4 (0.007)	3 (0.55)	1 (0.39)
Anti-ribosomal-P n=4 (10%)	3 (0.25)	0 (0.26)	2 (0.01)	4 (0.74)	3 (0.25)	2 (0.59)
Anti-Ro/La n=7 (17%)	4 (0.57)	0 (0.12)	5 (0.07)	7 (0.64)	3 (0.79)	3 (0.75)
Anti-phospholipid antibody n=2 (5%)	1 (0.94)	2 (0.01)	2 (0.63)	2 (0.82)	1 (0.94)	0 (0.26)
Anti-histone N=2 (5%)	1 (0.94)	0 (0.43)	1 (0.06)	1 (0.74)	0 (0.16)	0 (0.26)

All cases were treated with oral steroids and hydroxychloroquine. Due to severe disease activity at diagnosis, eight (20%) cases required high-dose methylprednisolone pulses. Intravenous cyclophosphamide was given to seven (17.5%) patients, whereas intravenous immunoglobulin was given to three (7.5%) in view of severe neurological involvement or macrophage activation syndrome. Both pulse methylprednisolone and cyclophosphamide were given in grade III/IV/V lupus nephritis and severe systemic illness. The most common steroid-sparing agent used was azathioprine in 29 (73%). Complete remission was achieved in 14 (35%) patients, whereas improvement was observed in 16 (40%) patients. A flare-up was seen in three (7.5%) patients, and the disease persisted in seven (17.5%) patients.

## Discussion

Females in the adolescent age group were predominantly affected in our study, similar to previously published literature. However, some adult studies have reported a very high female-to-male ratio in contrast to the pediatric population (23.9:1) [10]. These observations may be attributed to an estrogen surge during puberty and the post-puberty period. The mean age of presentation in previous studies is quite similar to our study [11,12].

Apart from constitutional symptoms, mucocutaneous manifestations were the most frequently observed systemic involvement, followed by hematological abnormalities, as evident from a study done by Uğurlu et al. [12]. These similar findings were also reflected in our cohort. In contrast to our study, Kini et al. and Mahmoud et al. demonstrated predominant renal involvement [3,13]. Our study demonstrated a high incidence of major organ involvement, affecting the renal, hematological, and CNS even at diagnosis. This finding is consistent with other pSLE studies but contrasts with adult-onset SLE cohorts, where such extensive organ involvement is less common at initial presentation [14,15].

One of the key determinants of poor outcomes in pSLE is lupus nephritis, which leads to end-stage renal disease. Renal involvement in our study is almost identical to the observations of Mondal et al. [6]. However, studies done by Srivastav et al. and Borromeo et al. demonstrated a higher incidence of renal involvement (65% and 67%, respectively) [16,17]. Class III and IV were the predominant histological findings among patients who underwent renal biopsies, similar to previous literature [14]. Low complements and high titers of anti-ds-DNA antibodies are often signs of a more aggressive disease and a higher risk of developing lupus nephritis. Monitoring these markers is crucial to managing SLE and preventing complications like lupus nephritis.

Kini et al. demonstrated hematological abnormalities in 81.3% of the children, with anemia (71.9%) being the most common [3]. A previous study from the United Kingdom reported hematological involvement in 91%, hemolytic anemia in 27%, leukopenia in 32%, and thrombocytopenia in 20% [18]. Our study echoes findings similar to both of the above studies. The prevalence of neuropsychiatric manifestations in pSLE varies from 20% to 50.9%, and the most common clinical features are headache, cognitive dysfunction, mood, and anxiety disorders [19]. We found CNS involvement in 22.5%. Another study from eastern India observed similar findings [13]. Three children were found to have unilateral vision loss during the initial course, which was reversible with the addition of systemic pulse methylprednisolone. Vision loss in SLE may be due to retinal vasculitis, brainstem infarction, anti-phospholipid antibodies, or hydroxychloroquine toxicity [20].

ANA positive was found in almost all our study participants, with two-thirds having a high titer of anti-ds-DNA antibody [1]. Consistent with past literature, we found that anti-DS-DNA antibody positivity had a strong relationship with both serositis and lupus nephritis [20]. Positive anti-phospholipid antibody (APLA) and antiribonucleoprotein antibodies were strongly correlated with neuropsychiatric manifestations in our study. While APLA is a well-established predictor of focal neurological symptoms in adult SLE patients, there is a dearth of research linking anti-ribonuceoprotein to neuropsychiatric manifestations [21].

Study participants were treated with a variety of modalities in varying combinations, depending on the severity of their condition and the involvement of organs. We achieved complete remission in 35% of patients. During three months of follow-up, three (7.5%) cases had a flare-up despite adequate immunosuppressive therapy. One of the earlier studies reported 59.4% of patients having flare-ups on long-term follow-up [3]. Fewer flare-ups in our study could be attributed to a good level of care and short-term follow-up. A smaller sample size and a lack of long-term follow-up are the limitations of our study.

## Conclusions

We have explored the complex realm of pSLE in our research, offering valuable perspectives on several facets of the illness's presentation, prognosis, and outcomes with findings comparable with Indian studies. Multicentric studies with long-term follow-ups are required in Eastern India to delineate the association of autoantibodies with organ-specific involvement and outcomes of pSLE.
